# A Role for Sigma Receptors in Stimulant Self Administration and Addiction

**DOI:** 10.3390/ph4060880

**Published:** 2011-06-17

**Authors:** Jonathan L. Katz, Tsung-Ping Su, Takato Hiranita, Teruo Hayashi, Gianluigi Tanda, Theresa Kopajtic, Shang-Yi Tsai

**Affiliations:** Psychobiology and Cellular Pathobiology Sections, Intramural Research Program, National Institute on Drug Abuse, National Institutes of Health, Department of Health and Human Services, Baltimore, MD, 21224, USA

**Keywords:** sigma receptors, drug abuse, cocaine, self-administration, reinforcing effects

## Abstract

Sigma_1_ receptors (σ_1_Rs) represent a structurally unique class of intracellular proteins that function as chaperones. σ_1_Rs translocate from the mitochondria-associated membrane to the cell nucleus or cell membrane, and through protein-protein interactions influence several targets, including ion channels, G-protein-coupled receptors, lipids, and other signaling proteins. Several studies have demonstrated that σR antagonists block stimulant-induced behavioral effects, including ambulatory activity, sensitization, and acute toxicities. Curiously, the effects of stimulants have been blocked by σR antagonists tested under place-conditioning but not self-administration procedures, indicating fundamental differences in the mechanisms underlying these two effects. The self administration of σR agonists has been found in subjects previously trained to self administer cocaine. The reinforcing effects of the σR agonists were blocked by σR antagonists. Additionally, σR agonists were found to increase dopamine concentrations in the nucleus accumbens shell, a brain region considered important for the reinforcing effects of abused drugs. Although the effects of the σR agonist, DTG, on dopamine were obtained at doses that approximated those that maintained self administration behavior those of another agonist, PRE-084 required higher doses. The effects of DTG were antagonized by non-selective or a preferential σ_2_R antagonist but not by a preferential σ_1_R antagonist. The effects of PRE-084 on dopamine were insensitive to σR antagonists. The data suggest that the self administration of σR agonists is independent of dopamine and the findings are discussed in light of a hypothesis that cocaine has both intracellular actions mediated by σRs, as well as extracellular actions mediated through conventionally studied mechanisms. The co-activation and potential interactions among these mechanisms, in particular those involving the intracellular chaperone σRs, may lead to the pernicious addictive effects of stimulant drugs.

## 1. Introduction

We recently reported on the reinforcing effects of sigma receptor (σR) agonists in rats that had a history of cocaine self administration. That rats would self administer a σR agonist was a surprising outcome. Several previous studies had demonstrated that σR agonists were ineffective in behavioral procedures that were indicative of reinforcing effects (see below). Thus, the present paper explores the finding of reinforcing effects of σR agonists further with regard to its implications for the role of σRs in the abuse of cocaine, drug abuse in general, adaptations to cocaine exposure, and the potential treatment of stimulant abuse.

There have been a number of comprehensive reviews that have focused on the potential of σR antagonists as treatments for stimulant abuse (e.g. [1-3]), There also are a number of reviews of the behavioral effects of various ligands for the σR ([e.g. [4,5]). The interested reader is referred to those papers for a more comprehensive overview and an introduction to the literature on the behavioral pharmacology of σRs.

The present paper will provide a brief overview of the history of research on σRs and the current understanding of the cell biology of σRs. We will then review the literature on σR agonists with a focus on effects indicative of abuse liability – e.g. effects on dopamine (DA) in brain regions critical for reinforcing effects, locomotor stimulation, subjective discriminative-stimulus effects, self administration, and place conditioning – in light of the finding that σR agonists can be self administered. Finally, we will further examine several results with cocaine focusing on σRs that may have implications for the reinforcing effects of σR ligands.

As the history of σR research clearly indicates, there have been a number of false starts, quirks, and obstacles due to the need for refinement of techniques and a better understanding of the ligands used to assess actions at σRs. Consequently, the present review will focus on ligands that are currently understood to be relatively selective for σRs and will only when necessary include studies conducted with drugs that were previously thought to be σR ligands but have been proven less selective.

### 1.1. History of σRs

The initial proposal by Martin and colleagues [6] was that σRs were a subtype of opioid receptor that were responsible for “psychotomimetic” effects of various opioid agonists, including the prototype agonist, SKF 10,047. However, subsequent studies indicated that these effects of SKF 10,047 were insensitive to blockade by opioid-receptor antagonists such as naloxone [7]. Due to cross-recognition of various putative σR ligands such as SKF 10,047 at σRs and the high-affinity PCP binding site located within the NMDA glutamate receptor complex [8], and similar behavioral effects of these drugs [9], confusion reigned. The subsequent identification and characterization of more selective ligands, including dizocilpine for the PCP site [10] and DTG for sigma sites [11] allowed for the pharmacological identification of σR sites that were unique from other known binding sites in the central nervous system (see [12] for a review). Pharmacological and molecular studies have distinguished two subtypes of σRs. The σ_1_R has been cloned and characterized as a 24 kDa single polypeptide having no homology with any other known mammalian proteins. In contrast, the σ_2_R is a 18-21 kDa protein that has not yet been cloned. Consequently, much more is known about the σ_1_R than the σ_2_R. Subsequent studies have indicated that σRs are expressed throughout the CNS and have been implicated in a variety of physiological functions and disease states [13].

### 1.2. Current Understanding of σR

#### 1.2.1. Cell Biology

##### Structure and molecular function of the σ_1_R

The σ_1_R is an integral membrane protein predominantly expressed at the endoplasmic reticulum (ER). The σ_1_R possesses two membrane-spanning domains at the N-terminus and the center of the protein [14]. The second transmembrane domain and the putative membrane-anchoring domain at the C-terminus are postulated to comprise a ligand-binding pharmacophore [15,16]. The σ_1_R shares no homology with any mammalian proteins, but shares 30% identity with a yeast C8-C7 sterol isomerase [17]. Interestingly, the second transmembrane domain of the σ_1_R shares over 80% identity with the sterol-binding pocket of sterol isomerase [17], supporting a notion that the σ_1_R is a sterol-binding protein utilizing the membrane-embedded domain for the association with lipid ligands.

**Figure 1:**
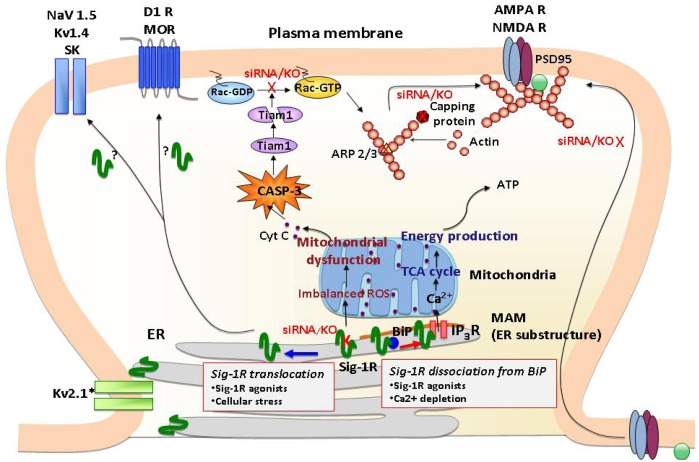
Potential synaptic actions of σ1Rs.

The C-terminus of the σ_1_R possesses chaperone activity that prevents protein aggregation [14]. It has been suggested that the chaperone domain resides in the lumen of the ER [14], thus σ_1_Rs stabilize ER lumenal and/or ER membrane proteins. The chaperone activity of the σ_1_R is regulated by a direct protein-protein interaction with another ER chaperone, binding immunoglobulin protein/78 kDa glucose-regulated protein (BiP/GRP-78) [14] (Figure 1). The striking characteristic of the σ_1_R is that the chaperone activity can be manipulated by synthetic or endogenous ligands or by cations such as Ca2+ in a clear agonist-antagonist manner [14,18] (Figure 1). The σ_1_R in complex with BiP is basically in the dormant state [14]. Upon binding of σ_1_R agonists or the depletion of Ca2+ in the ER, σ_1_Rs dissociate from BiP, thereby shifting to the active state [14,18]. In contrast, σ_1_R antagonists strengthen the association with BiP, thus inhibiting the action of agonists [14,18]. Accordingly, in living systems the chaperone activity is rapidly activated either by applications of σ_1_R agonists or by activation of IP3 receptors *via* Gq-coupled metabotrophic receptors at the plasma membrane [14]. Further, a recent study demonstrated that oxidative stress also regulates the association between BiP and σ_1_R [19]. Thus, a wide range of neuronal activities, which lead to oxidative stress or Ca2+ mobilization, including dopaminergic neural transmission, might contribute to the dissociation of the σ_1_R from BiP, though this possibility has not been tested *in vivo*.

##### Subcellular distribution of σ_1_Rs

Since the early proposal of the sigma “opioid” receptor [20], a large amount of research was directed at assessing actions of σ_1_Rs at the plasma membrane, particularly actions related to trimeric G proteins [21]. Nevertheless, no conclusive evidence had been provided to support the direct coupling of σ_1_Rs to plasma membrane G proteins. Following the successful cloning of σ_1_Rs [17], molecular biological or immunological approaches have been aggressively introduced in the research field to explore the molecular function and precise subcellular localization of σ_1_Rs. In terms of subcellular localization of σ_1_Rs, results from a line of recent studies are briefly summarized as follows: 1) The majority of σ_1_Rs localizes at the ER (including nuclear envelopes) in a variety of cells or organs; 2) σ_1_Rs tend to form clusters at ER membranes; 3) Unambiguous evidence supporting a significant level of σ_1_R accumulation at plasma membranes has not been convincingly provided; 4) The ER distribution and the level of σ1Rs can be dynamically or rapidly changed under certain conditions.

One of the major loci where σ_1_Rs cluster is the ER subdomain physically associating with mitochondria (mitochondria-associated ER membrane: MAM) [14] (Figure 1). The MAM is a center place where the ER directly provides Ca2+ to mitochondria *via* IP3 receptors and transports phospholipids and sterols to mitochondria [22]. The Ca2+ provided from MAM to mitochondria is known to activate the tricarboxylic acid (TCA) cycle and ATP synthesis [23]. σ_1_R chaperoning IP3 receptors at the MAM potentiates Ca2+ influx from the MAM to mitochondria [14], thus likely regulating mitochondrial bioenergetics and reactive oxygen species (ROS) generation (Figure 1).

A recent study demonstrated that σ_1_Rs are also highly clustered at the thin layers of ER cisternae adjacent to the post-synaptic plasma membranes of the ventral horn spinal motor neurons [24]. The post-synaptic clusters of σ_1_Rs are specific to cholinergic synapses [24]. Thus, in specific neuron types, σ_1_Rs are constitutively expressed at the ER subdomains apposing the plasma membrane (Figure 1). Similar plasma membrane clustering of σ_1_Rs was also observed in living NG108 neuroblastoma x glioma hybrid cells when enhanced yellow fluorescent protein-tagged σ_1_Rs were expressed [14].

##### Mobility and translocation of σ_1_Rs

The demand for elucidating the molecular mechanism by which σ_1_Rs regulate plasma membrane events is expanding as various novel roles are unveiled for σ_1_Rs in the regulation of G protein-coupled receptors (GPCRs) and ion channels [25-28]. σ_1_Rs tonically regulate activity of potassium, NMDA, and sodium channels [25,26,29] (Figure 1). Recent studies indicate possible interactions between σ_1_Rs and GPCRs, such as μ opioid and DA D1 receptors [27,28] (Figure 1). In light of the nature of molecular chaperones, studies suggest that σ_1_Rs regulate plasma membrane proteins *via* physical protein-protein interactions [25,27,28]. Although further studies are essential for clarification, growing evidence from recent molecular and cell biological studies is beginning to elucidate possible mechanisms that may in part explain plasma membrane actions of σ_1_Rs. Three potential mechanisms are proposed in following.

Cellular stress or σ_1_R agonists are shown to mobilize σ_1_Rs at the ER membrane [30,31] (Figure 1). The highly mobile σ_1_Rs move along the ER membranes from deep intracellular loci (e.g., MAM) to more peripheral subcellular locations [31]. At the MAM σ_1_Rs are highly stationary [31], possibly due to their tight association with cholesterol/ceramide-rich lipid microdomains therein [32]. However, over 70% of σ_1_Rs localized at non-MAM ER membranes (e.g., ER membranes in neurites) are highly mobile with a mobility speed that reaches around 8-10 μm/min [31,33]. Upon ligand binding, σ_1_Rs redistribute from detergent-insoluble lipid microdomains to soluble membrane domains [34,35]. σ_1_R agonists may unleash σ_1_R proteins from lipid microdomains, thus gaining mobility at the ER. The resulting peripherally distributed σ_1_Rs, as seen at cholinergic synapses of motor neurons [24], may be able to reach close proximity with the plasma membrane. Therefore, translocation may enable σ_1_Rs from the ER to physically associate with proteins at the plasma membrane (Figure 1).

Some ER chaperones are known to translocate from ER to other intracellular organelles, or be released to the outside of cells [36,37]. The mechanism underlying the translocation of ER chaperones involves the hindrance of ER retention/retrieval motif *via* protein-protein interactions [37,38]. The ER localization of σ_1_Rs seems to be determined by the double-arginine ER retention motif at the N-terminus that is utilized for a retrieval of ER proteins from coat protein complex-I (COP-I)-operated ER-Golgi secretory pathway to the ER. The deletion of the motif causes the exclusive relocation of σ_1_Rs from ER to the cytoplasm or cytosolic lipid droplet-like structures [34]. The wild-type σ_1_Rs are indeed co-immunoprecipitated with COP-I, indicating that σ_1_Rs are actively retrieved from the ER-Golgi secretory pathway to the ER. In contrast, mutations at the double-arginine motif disrupt the association of σ_1_Rs with COP-I [39]. A recent bioinformatics study identified that 84 mammalian membrane proteins possess the double-arginine motif in the first 25 amino acids [39]. Interestingly, only 24 of these proteins localize at the ER or sarcoplasmic reticulum [39]. Several proteins with the double arginine motif successfully escape from the ER retrieval machinery and reach plasma membranes that include the plasma membrane ATP-sensitive potassium channel (Kir6.1/2), and GABA_B_ receptor GB_1_ subunit [39]. The NMDA receptor NR1-1a subunit is also known to possess the triple-arginine ER retention motif [40]. Whether the interaction of σ_1_Rs with ion channel subunits or GPCRs at the ER may hinder the double-arginine motif of σ_1_Rs, thus triggering the departure of the complex for the plasma membrane is an untested, but intriguing possibility.

σ_1_Rs may also modulate plasma membrane proteins by controlling their folding and secretion at the ER level. Virtually all plasma membrane proteins are synthesized at the ER. Newly synthesized proteins are properly folded with the aid of ER molecular chaperones followed by entering the ER-Golgi secretory pathway for further modifications and the delivery to the final destination (e.g., plasma membrane) [41]. A recent study demonstrated that a σ_1_R agonist indeed potentiates the secretion of brain-derived neurotrophic factor (BDNF) from neuroblastoma cells [42]. The protein transport *via* the ER-Golgi secretory pathway is also controlled by lipids comprising the transport vesicles [43]. Cholesterol and sphingolipids which form lipid raft microdomains play a pivotal role in trafficking and sorting of plasma membrane proteins at the ER and Golgi [43]. Importantly, recent studies indicate that σ_1_Rs regulate lipid transport at the ER, and lipid raft formation at the plasma membrane [34,44,45]. These findings support a notion that σ_1_Rs may be involved in the transport of proteins as well as lipids between ER and plasma membranes. It should be mentioned that the transport of proteins from the ER to the plasma membrane is highly efficient, generally taking only a few to 30 min [41]. Specifically, protein delivery at dendritic spines is thought to be much faster because all machineries necessary for protein synthesis and trafficking are packed in the small structure [46,47]. From this viewpoint, it is plausible to speculate that σ_1_Rs may indirectly regulate the protein expression on the surface of neurons in a relatively short time frame by controlling protein transport.

##### σ_1_Rs and cellular morphologies

σ_1_Rs may affect a wide range of cellular functions by regulating cell morphologies. Earlier studies found that σ_1_Rs promote neurite sprouting in PC12 cells. Selective σ_1_R agonists, though having no effect of their own, were able to enhance nerve growth factor (NGF)-induced neurite sprouting. Additionally, NGF as well as chronic treatment with σ_1_R agonists were shown to up-regulate endogenous σ_1_R expression in PC12 cells; the process is essential for promoting neurite sprouting by σ_1_R ligands [48]. A later, similar finding of NGF-induced neurite outgrowth in PC12 cells revealed that the σ_1_R agonist SA4503 stimulates σ_1_R binding to IP3 receptors as well as the pathways downstream from trophic factor receptors that include PLC-γ, PI3K, p38 mitogen-activated protein kinase (MAPK), JNK and Pas/Raf/MAPK [49].

Recently, the impact of σ_1_Rs on synaptic plasticity and the consequential effects on neuronal function have begun to be elucidated. In σ_1_R deficient hippocampal neurons, aberrant morphologies have been observed [50]. σ_1_Rs are critical regulators for dendrite extension and branching during early stages of neuronal development. At later developmental stages when neurons are approaching maturation, σ_1_Rs facilitate the formation and maintenance of dendritic spines and functional synapses [50]. Thus, σ_1_Rs regulate both the early (e.g., neurite sprouting, dendrite extension, and dendrite branching) and late (e.g., spine maturation, synaptogenesis) stages of neuronal differentiation. When σ_1_Rs were depleted at the late stage of neuronal differentiation by siRNAs, neurons failed to form the mushroom-like spines as well as functional synapses that possess clustered assemblies of AMPA/NMDA receptors and postsynaptic density scaffolding protein PSD-95 [50] (Figure 1). The aberrant morphologies caused by σ_1_R depletion were associated with malfunctions of mitochondria followed by accumulation of ROS and activation of caspase-3 (CASP-3). In σ_1_R knockdown neurons, ROS-activated CASP-3 degrades T-lymphoma invasion and metastasis-inducing protein 1 (Tiam1) by proteolytic cleavages, thus subsequently reducing the active form of Rac1-GTP [50] (Figure 1). Both the mitochondria dysfunction and aberrant neuronal morphogenesis caused by σ_1_R knockdown were blocked by ROS scavengers, such as Tempol and N-acetylcysteine [50], indicating that σ_1_Rs are key modulators in maintaining the balance of oxidative stress in the neurons. A recent microarray analysis of rat primary neurons further demonstrated that the σ_1_R knockdown causes alterations of a cluster of transcripts involved in remodeling of the actin-based cytoskeleton network. The transcripts include those of actin capping proteins and actin-related protein 2/3 (ARP2/3) [51] (Figure 1). Together, these findings indicate that σ_1_Rs are important regulators in cellular morphology and neuronal plasticity.

#### 1.2.2. Receptor Binding

A wide variety of compound structures bind to the σR, which has made the study of structure-activity relationships difficult [52]. Obstacles to progress included difficulties in settling on the most selective radioligands for studies that were capable of differentiating the σR from PCP binding sites. The discovery of DTG as a selective σR ligand was a substantial advance in that regard [11]. In addition, the initial lack of appreciation that there are two types of σRs also impeded progress. The characterization of (+)-pentazocine as a prototype selective σ_1_R agonist was an equally important advance [53,54]. However, there are no ligands that can serve equally well as prototype selective σ_2_R agonists. Most commonly, DTG is used as a radioligand to label σ_2_Rs with adequate concentrations of unlabeled (+)-pentazocine to block the labeling of σ_1_Rs. Using these techniques more than adequate progress can be made in characterizing the binding of various ligands [55].

In a recent study primarily focused on the effects of σR agonists on DA neurotransmission, Garcés-Ramírez *et al.* [56] further characterized the binding of several prototype ligands at σ_1_ and σ_2_ binding sites using [^3^H](+)-pentazocine to label σ_1_Rs and [^3^H]DTG with cold (+)-pentazocine to label σ_2_Rs. The binding of the radioligands was consistent with previous descriptions. In addition, PRE-084, a ligand that selectively binds σRs over PCP binding sites [57], was reported to have affinity at σ_1_Rs approximately 600-fold greater than that for σ_2_Rs. Thus, PRE-084 can be used *in vitro* and *in vivo* as a selective σ_1_R agonist.

**Figure 2:**
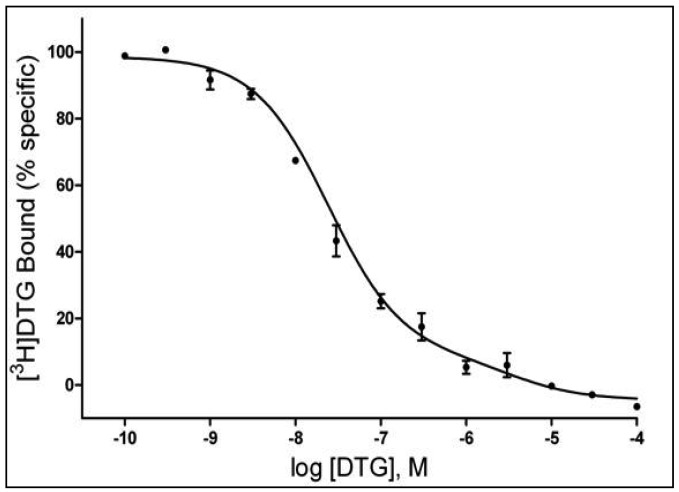
Homologous competition of radiotracer and nonradioactive DTG at guinea pig membranes.

A number of studies have suggested a multiplicity of binding sites beyond the sites commonly accepted as σ_1_ and σ_2_Rs [e.g. 58-60]. In the study by Garcés-Ramírez *et al.* [56], the binding data for [^3^H]DTG were better fit to a two-site than a single-site model. Figure 2 shows the results of a homologous competition study with DTG showing inflections in the displacement curve indicative of two binding sites with K_d_ values of 21.9 and 3,520 nM. The K_d_ value for the higher affinity site is comparable to that reported previously for DTG at σ_2_Rs. Additionally, the K_i_ values obtained with several compounds [56] further suggest that the DTG high-affinity site is the site recognized as the σ_2_R. In contrast, the low-affinity binding of DTG and the K_i_ values obtained for the other compounds studied were substantially below the values considered to be their σ_2_R affinities. As this binding was assessed in the presence of high concentrations of (+)-pentazocine (200 nM), the additional site appears to be a unique site different from the previously characterized σ_1_ and σ_2_R sites. Whether and how this low-affinity DTG binding site is related to previously identified multiple σR binding sites has not yet been determined.

#### 1.2.3. σR Agonists and Antagonists

What constitutes agonist and antagonist actions at σRs, and which ligands function in each of the two manners, have been significant problems throughout the history of research on σRs. The review by Walker *et al.* [52] describes much of the early research attempting to characterize various ligands with regard to their agonist or antagonist actions. Several factors hindered progress. Foremost is that the research approached these questions pertaining to the functionality of ligands at the binding site using a model based on GPCRs. As the sections above attest, the GPCR model is far enough from our current understanding of σRs to direct the research into less than profitable avenues. As described above, the chaperone activity of the σ_1_R can respond in clear agonist or antagonist modes, which can be assessed by its association with BiP. Agonists shift the receptor from the dormant state of BiP association to dissociation and antagonists strengthen the association with BiP, and inhibit the action of agonists [14,18]. How these molecular activities translate into *in vivo* actions remains unclear. Many of the compounds generally considered as σR antagonists are those that block the acute locomotor stimulant or toxic effects of cocaine, whereas agonists can shift the cocaine dose-effect curve leftward [12]. Cocaine binds to σRs, and itself “appears” to act as a σR agonist at high enough concentrations. Because of cocaine's pre-potent effects on DA systems, this method of identification remains somewhat less than satisfactory, but nonetheless successful.

### 1.3. Effects on Neurotransmitters

In regards to modulation of neurotransmitter release and possibly uptake, σRs have been shown to interact with GLU, ACh, DA, serotonin (5-hydroxytryptamine, 5-HT), norepinephrine (noradrenaline, NE), and γ-aminobutyric acid (GABA) systems. Some of these neuromodulatory actions could be of interest when related to reinforcing or dependence-producing actions of drugs, and will be described below.

#### 1.3.1. DA Neurotransmission

Several fundamental physiological functions have been related to DA neurotransmission, which notoriously underlies drug addiction as well as many different pathological conditions. Brain areas related to DA transmission express σRs [61], and some of the earliest functional studies indicated a modulation of dopaminergic effects by σR ligands [e.g. 62]. Thus, it has been with considerable interest that researchers have explored how activation or blockade of σRs modifies dopaminergic neurotransmission. Moreover, the discovery that stimulant drugs such as cocaine and methamphetamine bind to σRs has increased attention to σRs in studies of drug abuse.

As mentioned above, early difficulties in precisely delineating selective ligands for σRs have to be considered when examining contrasting results described in the literature. For example, (+)-3-PPP, a drug with high affinity for σRs, has been shown to significantly decrease extracellular DA levels in dialysates from the nucleus accumbens (NAC) [63] or from the striatum [64,65] after systemic administration. BMY-14802, a σR antagonist with 5-HT_1A_ agonist effects, attenuated the effect of (+)-3-PPP in the striatum. However, these effects of (+)-3-PPP are more likely the result of its agonist effects on DA D2-like receptors, which produce negative feedback on DA neurons, reducing firing and the release of DA [63].

A decrease in DA levels has also been shown with local administration of high doses of haloperidol, a σR antagonist, and the agonist, DTG [66,67]. A biphasic effect, an increase followed by a decrease in DA levels, has also been observed after intrastriatal administration of several σR ligands such as (+)-pentazocine, DTG, and (+)-MR200, a non-selective σR antagonist [66,67]. Moreover, systemic administration of DUP 734, (+)-SKF 10,047, and intrastriatal haloperidol, at low doses, increased DA levels [65,67], while DTG was without effects [65]. Increased DA turnover in the rat frontal cortex was also obtained after acute administration of SA4503, an effect that was blocked by the σR antagonist, NE-100 [68]. In addition, the effects of repeated administrations of SA4503, once a day for 21 days, was assessed in electrophysiological studies, in which it significantly increased the number of spontaneously active VTA dopaminergic neurons. Those electrophysiological effects were blocked by NE-100 as well [69].

**Figure 3:**
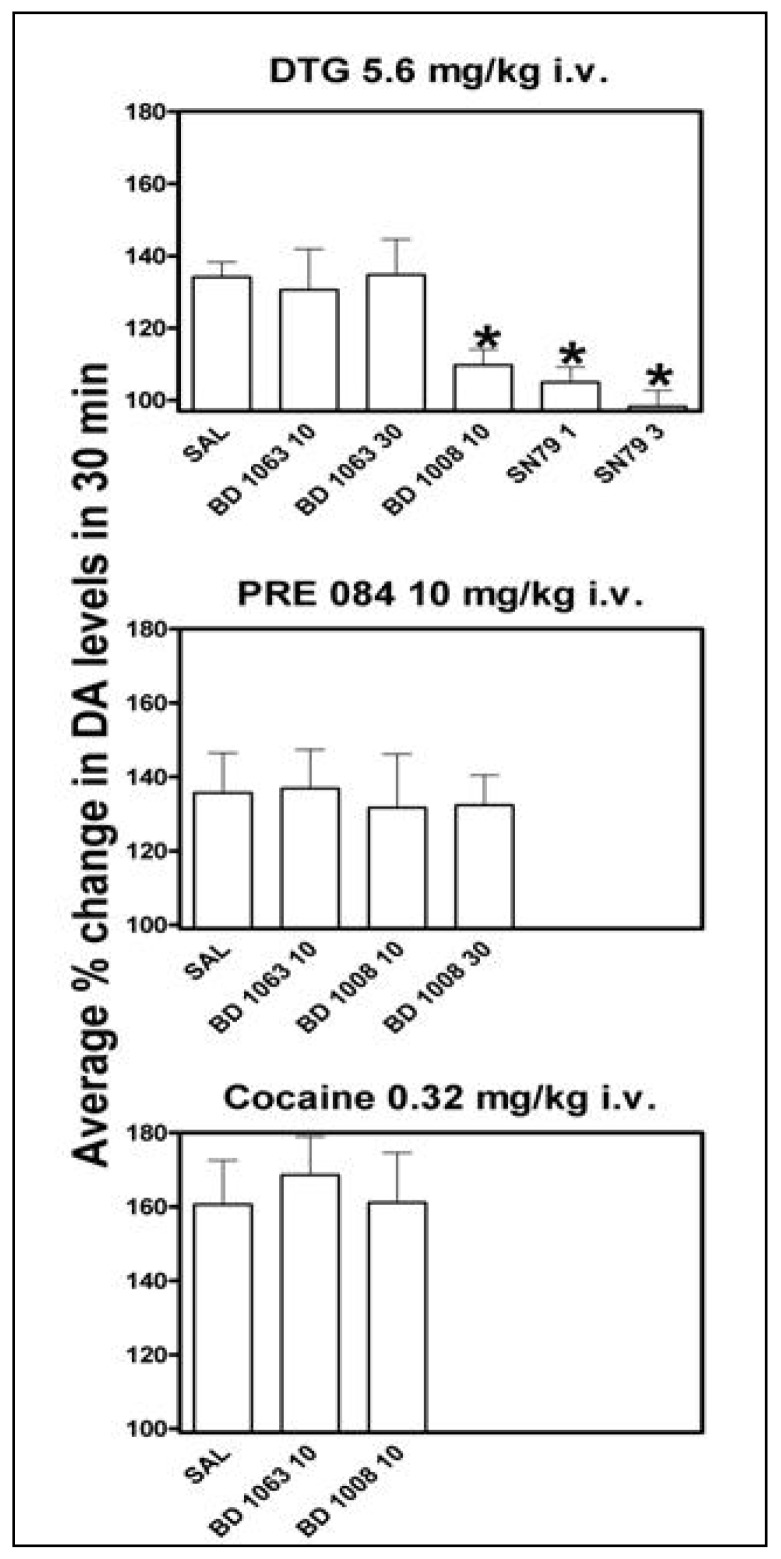
Effects of pretreatments with selective σR antagonists on DTG-, PRE-084-, and cocaine-induced stimulation of DA levels in dialysates from the NAC shell in rats.

In studies prompted by the finding of reinforcing effects of σR agonists [70], intravenously administered PRE-084 and DTG dose-dependently and significantly increased DA levels in the rat NAC shell [56], a brain area related to the reinforcing effects of drugs [71-73]. The effects of DTG were obtained at doses that approximated those that maintained self-administration behavior [70]. In addition, the effects of DTG were antagonized by BD 1008 (a non-selective σR antagonist) and SN 79 (a preferential σ_2_R antagonist) but not by BD 1063 (preferential σ_1_R antagonist), suggesting that the effect of DTG on DA was mediated by σ_2_Rs but not by σ_1_Rs (Figure 3, top). On the other hand, a significant increase in DA levels produced by PRE-084 was obtained at doses about 30 times higher than those that maintained self-administration behavior. Additionally, the effects of PRE-084 on DA were not significantly attenuated by pretreatments with σR antagonists (Figure 3, middle), suggesting the effects of PRE-084 on DA release were not mediated by σRs [56]. This dichotomy in the effects of PRE-084, with self-administration behavior mediated by σRs at doses that do not elicit increase in NAC shell DA levels (as compared to virtually all drugs abused by humans), and higher doses of PRE-084 that stimulate DA levels but are not mediated by σRs, suggest a DA-independent central pathway for σR-agonists reinforcing effects [56]. In the same report it was also shown that, in agreement with lack of effects in self administration studies [70], the acute cocaine-induced stimulation of DA levels was not affected by pretreatments with σR antagonists at doses able to produce full σR antagonist effects (Figure 3, bottom).

#### 1.3.2. GLU Neurotransmission

GLU neurotransmission, as with DA, has also been implicated in drug abuse, with its role purported to be in the various stages that lead from drug use to addiction [74]. Specific neuronal circuits in which GLU is the main neurotransmitter have been shown to be involved in neuronal adaptations that are induced by repeated exposures to abused drugs [75,76]. Modulation of GLU neurotransmission can be an important target of σR agonists. Moreover, GLU neurotransmission plays a role also in the modulation of the functioning of other neurotransmitter systems, thus its modulation by σR ligands can have a cascading greater influence than merely on GLU systems alone.

Several studies implicate σRs as influencing GLU neurotransmission in brain areas that play a role in learning and memory. For example, it has been reported that actions of neurosteroids at σ_1_Rs can enhance spontaneous GLU release in the prelimbic cortex and hippocampus [77,78]. In a report of an elegant series of experiments, Schiess and Partridge [79] suggested that another neurosteroid, pregnenolone sulfate, might act presynaptically through a Gi/o-coupled σ_1_-like receptor to modulate basal GLU release. Also, modulation of GLU release by dehydroepiandrosterone (DHEA) has been related to improved performance on an inhibitory avoidance task, possibly due to this neurosteroid's effectiveness in physiologically increasing GLU tone [80].

Recent evidence suggests that neurosteroids facilitate long-term potentiation (LTP) in the rat hippocampus, an effect suggested as being mediated by neurosteroid activation of neuronal σRs [29,81,82]. The pharmacological specificity of the effect has been shown by its prevention with co-administration of the selective σR antagonist, NE-100. As LTP has been implicated in adaptations concomitant with stimulant self administration [83] these findings have clear implications for stimulant dependence.

BDNF is a member of a family of growth factors found in brain and periphery that support the modification, growth, and differentiation of neurons and synapses. BDNF has been implicated in various processes related to learning and memory and is active in the hippocampus, cortex, and basal forebrain. It has also been implicated in the development of drug dependence [84-86]. Interactions between GLU release, BDNF, and σRs have been shown in studies of the effects of overexpression of σ_1_Rs on BDNF-induced PLC-γ activation and GLU release. In addition, BD 1047 prevents the potentiation of BDNF-induced PLC-γ activation and GLU release produced by antidepressant drugs [87].

#### 1.3.3. ACh Neurotransmission

σR agonists and neurosteroids with affinity for σRs have been shown to modulate ACh neurotransmission, and the stimulation of ACh release by σR agonists has been implicated in the improvement of cholinergic-deficit induced memory impairment. Thus σRs may play a role in modulation of learning and memory processes [88]. As preclinical and clinical studies have suggested the potential of cognitive enhancers in the treatment of stimulant dependence [89] a modulation of ACh systems by σR ligands may present another avenue for the discovery of treatments for stimulant dependence.

Igmesine potentiated the KCl-evoked release of [^3^H]ACh from rat hippocampal slices, an effect also produced by (+)-SKF 10,047, but not by DTG. Perfusion of the slices with the σR antagonist, haloperidol, blocked the effects of igmesine and (+)-SKF 10,047 [90]. Using brain microdialysis in rat prefrontal cortex, several σR agonists including (+)-3-PPP, DTG, (±)-pentazocine, and (+)-SKF 10,047 dose-dependently increased the extracellular ACh levels. The effect was antagonized by haloperidol [91]. Stereoselectivity and antagonism by haloperidol were also demonstrated in the stimulation of hippocampal extracellular ACh levels and anti-amnesic effects of (+)-SKF 10,047 [92].

The non-selective PCP/σR ligand, (+)-SKF 10,047, also stimulated ACh overflow in hippocampal slices [93] with a potency greater than that of DTG. In the same study, the striatal extracellular ACh levels were modestly increased by (+)-SKF 10,047, while DTG was without effects. Similar regional specificity for ACh stimulation was found with SA4503, a selective σ_1_R agonist, with effects in the rat frontal cortex and hippocampus dialysates and without significant effects on striatal ACh release [94,95]. It is also interesting to note that some neuroactive steroids, pregnenolone, DHEA [96], elicit an overlapping pattern of stimulation of ACh release [97], thus acting as σR agonists.

As mentioned above, cocaine-induced place conditioning has been blocked by σR antagonists [98,99] but not cocaine self-administration [70,100]. The blocking effects of σR antagonists on place conditioning procedure could result from the inhibition of ACh neurotransmission (amnesic action) rather than a specific antagonism of the effects of cocaine.

#### 1.3.4. NE Neurotransmission

The major abused psychostimulants, cocaine and amphetamines, increase NE neurotransmission after systemic administration [101], an effect which might play a role in the behavioral actions of these drugs [102]. Several σR ligands have been shown to interact with NE neurotransmission. The effects described below of (+)-3-PPP and haloperidol should be considered with the fact that these drugs also have activity at DA receptors.

The σR agonists (+)-pentazocine, BD 737, and DTG, inhibited, while other σR agonists, igmesine and (+)-3-PPP, facilitated NMDA-evoked overflow of [^3^H]NE from hippocampal rat slices, without affecting the basal efflux [103,104]. Antagonists of σRs, such as DUP 734, BD 1008, and haloperidol prevented both the facilitation and inhibition of the effects of σR agonists [103,104]. It was also demonstrated that σ_2_Rs might contribute to the regulation of NE release, since a σ_2_R antagonist, BIMU-8, reversed a σR agonist component of the effect that was not sensitive to σ_1_R antagonists [105]. Neurosteroids that activate σRs have been shown to differently modulate the K+-evoked, and NMDA-evoked, release of [^3^H]NE from hippocampal slices. For example, DHEA sulfate facilitated, while pregnenolone sulfate inhibited NMDA-evoked release of [^3^H]NE, and these effects were prevented by administration of σR antagonists [106]. Results from the latter study suggest that DHEA sulfate acts as a σR agonist, and further suggested to the authors that pregnanolone sulfate acts as an inverse agonist. Also, progesterone sulfate mimicked the antagonist effects of haloperidol, suggesting it might also possess antagonist actions at σRs [106].

#### 1.3.5. 5-HT Neurotransmission

It is interesting to note that several compounds initially believed to selectively target 5-HT neurotransmission possess affinity for σRs, and that this feature may play a role in the rapid onset of antidepressant efficacy, as compared to antidepressants that do not show σR activity [107]. 5-HT systems have also been implicated in various aspects of drug abuse. As with NE systems, many of the most avidly abused stimulant drugs interact with 5-HT neurotransmission, and neurotoxicity caused by several of these drugs impacts 5-HT brain systems [108]. Further σR ligands can modulate 5-HT neurotransmission, suggesting their involvement in the neurotoxic effects of amphetamines. For example, it has been suggested that DHEA by activating σ_1_Rs can negatively modulate 5-HT_3_ receptor activity in pyramidal cells of the pre-limbic cortex. This effect, in turn, has been shown to inhibit 5-HT-evoked GLU-release, which is mediated by activation of 5-HT_3_ receptors [109]. A pharmacological selective role for σ_1_ receptors in this effect has been shown mimicking the effects of DHEA with carbetapentane, a σ_1_ agonist, and blocking the effect with AC915, a σ_1_ receptor antagonist [109]. Another selective σR antagonist, MS-377, when injected alone had no significant effects on 5-HT or DA release in the rat striatum or in the medial prefrontal cortex. However, pretreatment with MS-377 significantly attenuated the behavioral effects of PCP, likely through the inhibition of PCP-induced increases in DA and 5-HT release [110].

Several studies demonstrated an effect of σR agonists on the firing of 5-HT neurons in the dorsal raphe nucleus (DRN). For example, a complex relationship between σR agonists and 5-HT neurotransmission has been shown by Bermack and Debonnel [111]. Using extracellular *in vivo* recordings in anaesthetized rats (+)-pentazocine and 4-IBP, but not PRE-084 or igmesine, markedly increased 5-HT firing after 2 or 21 days of treatment. In addition, the selective σ_1_R antagonist, NE-100, blocked the effects of (+)-pentazocine but not those of 4-IBP. The authors hypothesized the existence of subtypes of σ_1_Rs to explain the different results obtained with the different σ_1_R agonists. Because increased 5-HT neurotransmission might be implicated in neurotoxicity induced by some psychostimulants, like MDMA and methamphetamine, for example, it is worth noting that a recent paper shows that pretreatment with the non-selective σR antagonist, AC 927, significantly attenuated methamphetamine-induced striatal 5-HT depletions, striatal 5-HT-transporter reductions, and hyperthermia [112]. These results strongly suggest that blockade of σRs can alter 5-HT neurotransmission and this action might protect against methamphetamine-induced neurotoxicity.

#### 1.3.6. GABA Neurotransmission

Pregnenolone and (+)-SKF 10,047 inhibit the GABA-dependent inhibitory postsynaptic currents in rat hippocampal cell cultures [113], and the effects were antagonized by haloperidol and BD 1063. The effects were also blocked by pertussis toxin, suggesting a presynaptic location of σRs and their coupling with Gi/o proteins [113]. Because the hippocampus has a role in learning and memory, interactions of σR and GABA systems such as those demonstrated by Mtchedlishvili and Kapur [113] suggest that circulating neurosteroids with affinity for σRs might be involved in modulation of learning and memory [see 114]. In general, the inhibition of spontaneous release of GABA might facilitate release of other neurotransmitters throughout the CNS, altering the function of other neurotransmitter systems.

## 2. Cocaine and σRs

### 2.1. Binding Studies and Acute Toxicity

Several years ago Sharkey *et al.* [115] reported that cocaine had affinity for σRs. The affinity of cocaine was determined with [^3^H]haloperidol using 25 nM of unlabeled spiperone to block the labeling by the radioligand of 5-HT_2_ and D2 receptors. The affinity of cocaine for the σR was reported to be 6.7 μM. More recently the affinity of cocaine for σ_1_ and σ_2_ receptors was reported with the current conventional radioligands and assay conditions for these sites [56]. The affinity of cocaine for the σ_1_R using [^3^H](+)-pentazocine was reported to be 5.19 μM. In addition, the affinity of cocaine for the σ_2_R was reported to be 19.3 μM using [^3^H]DTG with excess (+)-pentazocine (200 nM) to block the binding to σ_1_Rs.

Sharkey *et al.* [115] argued that concentrations in brain sufficient to bind to σRs would be reached at high doses of cocaine that produce acute psychotic reactions in humans. However, the authors also argued that actions at σRs were not likely to contribute to the reinforcing effects of cocaine that are obtained at lower doses. Seemingly consistent with the findings of Sharkey *et al.* a multiple regression analysis among potencies of cocaine-like agents to produce seizures or lethality indicated that a substantial amount of the variance for either effect was accounted for by binding to the 5-HT or DA transporters, respectively. However, σR binding as well as binding at muscarinic sites appeared to attenuate seizure-producing or lethal effects of the cocaine-like drugs [116].

Several studies have examined more closely the interactions between cocaine and a large number of σR ligands [see review by 1]. For example, the σR antagonists, BD 1047 and LR172 blocked cocaine-induced seizures, lethality, and locomotor stimulation [117]. Other studies have found that the σR agonist, DTG, enhanced the convulsive effects of low doses of cocaine that had no convulsive effects when administered alone and lowered the cocaine LD_50_ value [118]. Finally, antisense directed at area -97 to -77 after the initiation codon of a cloned cDNA sequence for σ_1_Rs from mouse was injected *via* indwelling cannulae to the lateral ventricles. Three infusions of 10 μg/5 mL were administered over a four-day period. This antisense treatment attenuated the convulsive and locomotor stimulant effects of cocaine whereas a mismatch sequence was relatively less active. In addition, the treatment decreased the B_max_ for σR binding by 38 to 45%. The functional pharmacology therefore suggests that increased σR activity enhances, whereas decreased σR activity either by antagonist or antisense treatment, attenuates the effects of cocaine. Those functional results are difficult to reconcile with the statistical approach of Ritz and George [116] that suggests that the actions of cocaine-like compounds at σRs diminish the seizure-producing and lethal effects of cocaine. Cocaine has been fairly well substantiated as a σR agonist [12,14]. Whether the other “cocaine-like” compounds examined by Ritz and George [116] have σR agonist or antagonist effects has not been established.

### 2.2. Locomotor-Stimulant Effects

The first study that established that σR antagonists could block the locomotor stimulant effects of cocaine [119] examined the effects of BMY 14802 and rimcazole, and compared their effects to those of clozapine, haloperidol, and (+)-3-PPP. Both of the σR antagonists blocked the locomotor stimulant effects of cocaine at doses that were inactive when administered alone. In contrast, the antagonism of the locomotor stimulant effects of cocaine produced by the DA receptor antagonists required doses that also decreased activity when administered alone (see also [120]). Similar results were reported by Okuyama *et al.* [121] with methamphetamine induced locomotor activity. Interestingly, the selective σR antagonist, NE-100, was inactive in the latter study. Nonetheless, a substantial literature has indicated that antagonism of the locomotor effects of stimulant drugs is produced by a wide variety of selective σR antagonists, such as BD 1063, and that the antagonism is similar to that produced for convulsions and lethality [118,122]. In addition, several σR antagonists significantly attenuated the development of cocaine-induced locomotor sensitization [123,124]. The selective σ_1_R antagonist MS-377 also attenuated the sensitization to stereotyped behavior induced by methamphetamine [125].

The antagonism of locomotor effects of stimulant drugs poses the question of whether σR agonists have stimulant effects of their own. The selective σ_1_R agonist, (+)-pentazocine (1.0 to 10.0 mg/kg), did not affect locomotor activity in mice [126]. However, another study indicated relatively modest increases in locomotor activity produced by 32 mg/kg of (+)-pentazocine in rats [127]. Another selective σ_1_R agonist, PRE-084, has been reported to have no effect on locomotor activity in mice up to doses of 10 [128] and 60 mg/kg [129]. The nonselective σR agonist, DTG has been studied at doses up to 20 mg/kg and only decreased activity at the highest dose tested [130]. Despite its lack of locomotor stimulant effects of its own, DTG potentiated cocaine induced locomotor stimulant effects in rats [131]. Similarly, the σR agonist, SA 4503, which has been reported to be ∼14- [132] to ∼100-fold [133] selective for the σ_1_R over the σ_2_R, only decreased locomotor activity in rats across the range of behaviorally active doses [134,135]. Thus it appears that σR agonists are relatively devoid of locomotor stimulant effects.

### 2.3. Discriminative Stimulus Effects

Several studies have examined the discriminative-stimulus (subjective) effects of σR agonists. In these studies, the subject is trained using operant conditioning techniques to emit one response (intermittently reinforced typically with food pellets) after administration of vehicle and a different response after administration of a behaviorally active drug. Once subjects are trained to some level of accuracy, typically greater than 90% of responses appropriate to the vehicle and “training” drug conditions, they can be tested with various treatments to assess the degree to which the treatment produces or modifies the discriminative effects of the training drug (see [136] for a full description of the technique). Typically drugs that share pharmacological mechanism with the training drug will produce a response from the subject similar, if not identical, to (>90% training drug-appropriate responding) the training drug. For example, in rats trained in a cocaine-discrimination procedure, the cocaine analog, WIN 35,428, which has approximately 10-fold higher affinity than cocaine for the DAT is 10-fold more potent than cocaine and at the appropriate dose produces ∼100% cocaine-appropriate responding [e.g. 137].

Studies of the discriminative stimulus effects of σR agonists were hampered initially by lack of clear indications of which compounds had the selectivity necessary to serve as a standard against which other compounds could be compared. Initial studies with dextromethorphan [138,139] and (+)-SKF 10,047 [140,141] did not distinguish between phencyclidine (PCP)-like compounds and σR agonists. Singh *et al.* [142] examined the potential of several compounds with high affinity for the σR to displace [^3^H]DTG from CNS sites in *ex vivo* binding assays and to substitute for the discriminative effects of (+)-SKF 10,047. Racemic pentazocine (with a concomitant dose of naloxone to block its opioid effects) substituted for (+)-SKF 10,047, however the other compounds that displaced [^3^H]DTG were ineffective either as agonists or antagonists of (+)-SKF 10,047. In addition substitution was obtained with MK-801, a PCP receptor ligand with no appreciable affinity for the σR. The authors concluded that their results were consistent with an NMDA-receptor mediation of the discriminative-stimulus effects of (+)-SKF 10,047 and that there was no evidence for a role of σRs in the discriminative stimulus effects of (+)-SKF 10,047. However, the results with pentazocine/naloxone combinations suggest that the conclusions be reconsidered.

Rats trained to discriminate between s.c. injections of DTG (3.0 mg/kg) and saline generalized fully or virtually fully to PCP and related drugs as well as various opioid receptor agonists and (+)-enantiomers of benzomorphans [143]. Additionally, a group of rats trained to discriminate saline from 2.0 mg/kg of PCP generalized completely to DTG. Thus the selectivity of DTG as a σR radioligand, does not carry over to the behavioral effects of the drug. The disconnect between radioligand binding and behavioral outcomes requires further study.

Steinfels *et al.* [144] trained rats to discriminate 2.0 mg/kg of (+)-pentazocine from saline injections (s.c.). In studies of substitution, (+)-SKF 10,047 substituted fully for the training dose of (+)-pentazocine, which is consistent with radioligand binding studies that have indicated cross recognition of (+)-SKF 10,047 at PCP and σR sites. However, PCP itself, which has low affinity for σR sites, did not substitute for the training dose of (+)-pentazocine up to a dose of 6.0 mg/kg which had grossly observable pharmacological effects. This study, though limited, suggests that the discriminative-stimulus effects of (+)-pentazocine are different from those of PCP and further that this compound shows the most promise as a selective σR agonist for *in vivo* studies.

In rats trained to discriminate s.c. injections of cocaine (10 mg/kg) from saline, DTG (1 and 10 mg/kg, 30 min before sessions) was no different from vehicle [145]. Similar results were reported by Cohen and Sanger [146] in abstract form. Additionally, the σ_1_R agonist, SA 4503, fully substituted for neither cocaine nor methamphetamine in rats trained with food reinforcement [134,135]. More recently both PRE-084 and DTG were examined in rats trained to discriminate cocaine (10 mg/kg, i.p.) from saline [147]. Neither compound substituted for cocaine when administered i.p., s.c. or i.v., 5 or 30 min before testing. In contrast, the standard DA uptake inhibitors, WIN 35,428 and methylphenidate, both fully reproduced cocaine-like discriminative-stimulus effects, as has been shown previously [137,148,149].

Other drug-discrimination studies demonstrated that the σ_1_R agonist, (+)-pentazocine, fully substituted in rats trained to discriminate ethanol [150] or buprenorphine [151]. These results suggest caution in the universal acceptance of (+)-penatzaocine as a selective σ_1_R ligand. However, the results are consistent with the suggestion of little, if any, overlap of the discriminative-stimulus effects of σR-agonists and stimulant compounds and therefore that the reinforcing effects of the σR agonists [70] are not based on substantial overlap of the subjective effects of the two classes of drugs.

### 2.4. Place Conditioning

Place conditioning, or conditioned place preference as it is commonly known, is a procedure in which a drug is administered to the subject in a particular environment which typically consists of distinct tactile, visual, or olfactory stimuli. Vehicle is injected when the subject is in a different environment. The two environments are virtually always separate sections of a larger chamber. After several pairings of the environments with the respective injections (conditioning) the subject is given a test of the effectiveness of the conditioning by allowing it unrestricted access to either environment without drug administration. Virtually all of the commonly abused drugs produce a shift in the amount of time allocated within the two sections of the chamber such that the subject spends more time in the section paired with the drug [152].

An increase in time allocation compared to before conditioning is considered by many to be a measure of the reinforcing effect of the drug in place conditioning. It should be noted that this is a Pavlovian (or respondent) conditioning procedure because two sets of stimuli are paired: the stimuli that arise from being in the particular section of the chamber and those from the drug injection. In Pavlovian conditioning the pairing of two stimuli is considered reinforcement. This type of conditioning contrasts with self-administration procedures which involve operant conditioning in which a response of the subject is paired with a stimulus. In the self-administration procedure following a response with a stimulus, in this case those from the drug that is injected, is reinforcement if the response increases in probability. It is perhaps unfortunate, and potentially confusing, that the two procedures use the same term, reinforcement, to specify its inherent process. It may be a further source of confusion that in the place conditioning procedure the final test of the conditioning involves entry into a section of the chamber, which can be considered an operant response with the stimuli arising from entry into that section of the chamber as its consequence. It is no surprise that the two procedures, operant and Pavlovian conditioning, are comingled and difficult to un-entangle, even in the laboratory.

Several σR antagonists have been shown to block place conditioning produced by stimulant drugs, particularly cocaine. This effect was first reported by Romieu *et al.* [98] using mice. In that study, the σR antagonists, NE-100 and BD 1047 (each at 1.0-10.0 mg/kg), dose-dependently blocked the place conditioning produced by cocaine (20 mg/kg) when administered in combination with cocaine during conditioning trials. The antagonists had no effects when administered alone. Additionally, *in vivo* administration of antisense oligodeoxynucleotides directed at the σ_1_R blocked place conditioning by cocaine whereas a mismatch oligodeoxynucleotide was inactive.

In another study with mice [99], the effects of NE-100 and BD 1047 were replicated and extended to conditioning produced by another DAT inhibitor, BTCP. In addition to blocking the conditioning of a place preference, NE-100 (3.0, 10.0 mg/kg) and BD 1047 (1.0-10.0 mg/kg) administered only on the post-conditioning test day decreased the amount of time spent on the cocaine-paired side of the chamber compared to that found with subjects given vehicle. Further, the σR agonists, igmesine and PRE-084 (each at 10.0-60.0 mg/kg), were examined for their potential to produce place conditioning when administered alone. Neither of these σR agonists had effects different from those of vehicle. The σR agonist, SA 4503 studied at 3.0 mg/kg, i.p. [153] and (+)-SKF 10,047 at doses from 1.0 to 4.0 mg/kg [154] were also inactive in producing a place conditioned effect in rats.

The neuroactive steroids, DHEA (5.0-20.0 mg/kg, s.c.) and pregnenolone (10, 20 mg/kg, s.c.) were similarly inactive with regard to the induction of place conditioning [155]. Further, progesterone (10-40 mg/kg, s.c.) antagonized the place conditioning produced by cocaine (20 mg/kg, i.p.). Progesterone is a σR antagonist whereas DHEA and pregnenolone are σR agonists. Of course, as the authors noted, in addition to activity at σRs each of these neuroactive steroids has effects on other systems. Nonetheless, these results are consistent with the general observation that σR antagonists block the place conditioning induced by stimulant drugs but that σR agonists themselves are ineffective for the induction of place conditioning.

The place conditioning data suggest that actions mediated by σRs are necessary for cocaine-induced place conditioning. However, as the agonists were inactive, activation of σRs alone is not a sufficient condition for place conditioning. The results with place conditioning are similar to results described above for the stimulant-induced stimulation of locomotor activity. The σR antagonists were effective in blocking both locomotor stimulation and place conditioning produced by stimulant drugs. On the other hand, σR agonists by themselves were inactive in inducing the effect that their antagonists were effective in blocking.

### 2.5. Self Administration

In an early study, Slifer and Balster [156] compared the reinforcing effects of the stereoisomers of the 6,7-benzomorphans, SKF 10,047 and cyclazocine, to those of PCP in rhesus monkeys trained to self administer cocaine under an fixed ratio (FR) 10 schedule of reinforcement. Neither the racemic forms nor the (−)-enantiomers of SKF 10,047 or cyclazocine were self administered at rates of response greater than those maintained by vehicle. In contrast, both (+)-SKF 10,047 and (+)-cyclazocine were self administered, with the maximal response rates maintained comparable to those maintained by either PCP or cocaine. At the time of the study, the distinction had not yet been conclusively made between σRs and the PCP binding site. Because the drugs were not selective, the effects obtained were interpreted in terms of PCP-like effects of the (+)-enantiomers. Nonetheless, it remains possible that agonist actions at σRs may have contributed to the self administration of both (+)-SKF 10,047 and (+)-cyclazocine. Because cocaine appears to act as an agonist at σRs, a reinterpretation of these findings as involving σRs suggests that the reinforcing effects of cocaine could be altered by σRs antagonists.

More recently Martin-Fardon *et al.* [100] examined the effect of BD 1047 on cocaine self administration in rats. In that study, BD 1047 pretreatment did not affect cocaine self administration under an FR 5-response schedule, and was similarly ineffective on responding reinforced with sweetened-condensed milk in another group of rats. The authors also examined the effects of BD 1047 under a “reinstatement” procedure. This procedure has become popular as several authors have asserted that it is a valid model of relapse to drug taking [for a discussion of the merits of the assertion see 157]. Under the reinstatement procedure after training subjects to self administer cocaine the responding is then extinguished (responses no longer produce cocaine injections). Subsequently, conditions are imposed that increase the response rates above the low levels achieved during extinction (reinstatement). In the study by Martin-Fardon and colleagues, a stimulus previously associated with availability of cocaine self administration and absent during extinction was present during tests of “reinstatement.” BD 1047 dose-dependently decreased reinstated response rates with significant effects at 20 and 30 mg/kg. In contrast, reinstatement of responding previously reinforced with sweetened condensed milk was only decreased at 30 mg/kg. The authors suggested that their findings support a role for σRs in regulating conditioned responses to cocaine-related stimuli and further suggest these receptors as a potential target for the development of drugs to treat cocaine abuse.

**Figure 4:**
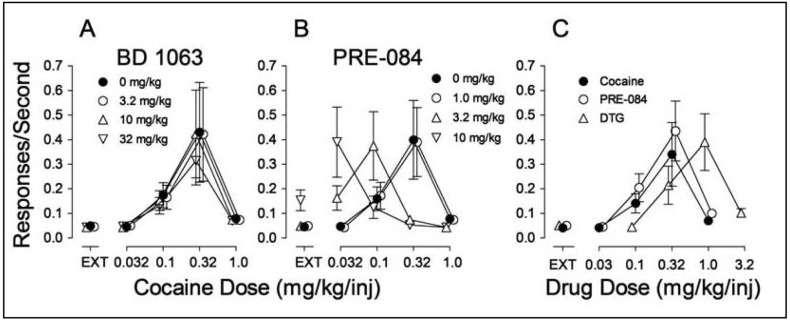
Reinforcing effects of cocaine, its modification by σR agonists and antagonists, and their substitution in rats trained to self administer cocaine.

In examining the potential involvement of σRs in the effects of cocaine we found, as in the study by Martin-Fardon *et al.* [100] that pretreatment of rats with σR antagonists had no effect on cocaine self administration [70] (Figure 4A). However, pretreatment with σR agonists produced a leftward shift in the cocaine dose-effect curve (Figure 4B). As these results resembled those obtained previously with standard DA uptake inhibitors, such as methylphenidate [158], it suggested that σR agonists may have reinforcing effects of their own. Figure 4C shows that the σR agonists, PRE-084 and DTG substituted for cocaine in rats trained to self administer cocaine, whereas σR antagonists did not (data not shown).

That the σR agonists would maintain self-administration behavior was surprising nonetheless. Though the study in monkeys by Slifer and Balster [156] had shown that the (+)-enantiomers of 6,7-benzomorphans would maintain self administration, that finding was interpreted as due to the PCP-like effects of the compounds. However, both DTG and PRE-084 are selective for σRs over the PCP binding site indicating that the effect, at least in the study by Hiranita *et al.* [70] was due to their actions at σRs. In addition, the self administration of the σR agonists was antagonized by each of the σR antagonists (BD 1008, BD 1047, BD 1063) studied, further substantiating that the self administration of the agonists was mediated by actions at σRs.

The antagonism of the self administration of PRE-084 and DTG by the σR antagonists also sheds light on other important aspects of the pharmacology of the σR system. As mentioned above, it has been difficult to clearly establish what can be considered as an agonist or antagonist effect of a σR ligand in preparations other than those involving σR chaperone activity. The self-administration studies show *in vivo* actions that can be useful in characterizing the agonist or antagonist effects of σR ligands. Additionally, the potency with which BD 1008, BD 1047 and BD 1063 blocked the self administration of the σR agonists is illustrative. In the paper by Martin-Fardon and colleagues described above, a dose of 20 mg/kg of BD 1047 was necessary to block the reinstatement of cocaine self administration. That dose is approximately 10-fold greater than the dose necessary to block the self administration of either PRE-084 or DTG, suggesting caution in interpreting the reversal of the reinstatement as a σR antagonist effect. Finally, the σR antagonists were all active as antagonists of PRE-084 and DTG self administration, but not cocaine self administration. Those findings suggest that the σR is not involved in the primary effect of cocaine that leads to its abuse. This latter consideration is consistent with findings that actions at the DA are the key components for the reinforcing effects of cocaine.

## 3. Effects of Chronic Cocaine Exposure

It is useful to consider the differences between outcomes in the place conditioning and the self-administration procedures. Most drugs that are abused (e.g. cocaine) are active in both procedures. The frequent consistencies between outcomes in the two procedures lead many to consider them as interchangeable predictors of abuse liability, with the underlying assumption that similar mechanisms are involved in both procedures. However, as detailed above σR antagonists block the acquisition of a cocaine-induced place conditioning (e.g. [98]), as well as the preferential time allocation after it is established (often referred to as its “expression,” e.g. [99]). In addition, σR agonists are inactive in producing place conditioning (e.g. [99]). In contrast, σR antagonists are inactive in blocking or otherwise altering the self administration of cocaine whereas σR agonists are self administered, at least in subjects with a history of cocaine self administration [70]. These differences reveal that self administration and place conditioning are not simply two procedures providing a “read out” of the same underlying reinforcement circuitry.

With specific regard to the σR influences on place conditioning during its acquisition, the analogous experiment with cocaine self administration has not been conducted. In the two studies of σR antagonists conducted to date the cocaine self administration was acquired prior to the testing. Nonetheless, the implication of the place conditioning studies is that there is a σR involvement in some adaptation to the repeated administration of cocaine, suggesting studies of alterations of central function induced by cocaine and related to σRs may shed light on cocaine abuse and chronic dependence.

### 3.1. Gene Regulation and Transcription

As described above, the acquisition of cocaine-induced place conditioning in mice was attenuated by co-treatment with σR antagonists [98,99,155]. In the 2002 paper [99], Romieu *et al.* also examined the effect of cocaine exposure on σ_1_R mRNA expression in the nucleus accumbens, caudate putamen, prefrontal cortex or cerebellum immediately after the subjects were tested for place conditioning. Comparative reverse transcriptase-polymerase chain reaction (RT-PCR) indicated a significant increase in σ_1_R mRNA occurred after the cocaine treatment in the nucleus accumbens but not in the caudate putamen, prefrontal cortex, or cerebellum. The authors suggest that the change in σ_1_R expression is integral to the reinforcing effects of cocaine as evidenced in the place conditioning procedure.

Several studies have examined changes in various substrates induced by cocaine, which may be sensitive to modification by actions at σRs. Matsumoto *et al.* [1] reported on microarray studies of gene expression in mice 20 min following administration of cocaine (at a dose that stimulates locomotor activity), BD 1063 (at a dose that blocks the cocaine effect) or the combination of the two compounds. Significant changes were confirmed using RT-PCR. Cocaine produced an up-regulation of 20 and down-regulation of 16 genes compared to tissue from non-treated mice. Of those, the σR antagonist blocked cocaine-induced changes in three genes: fos-related antigen 2 (fra-2), GPCR 27, and ataxia telangiectasia murine homolog. The authors suggested that the changes in fra-2 are noteworthy because cocaine stimulates the expression of other fos-related transcription factors. Because fra-2 is stimulated by second messengers the authors reasoned that the cocaine antagonist effects of BD 1063 might be due to its blockade of the activation of the second messengers induced by cocaine. In further studies Liu *et al.* [159] used microarray techniques to assess the effects of cocaine and BD 1063 on the expression of six fos and jun genes: fra-2, c-fos, fosB, c-jun, junB and junD. Cocaine increased the expression of fra-2 and junD, but only the alteration in fra-2 was blocked by co-administration of BD 1063. Further, the increases in fra-2 were followed by a later up-regulation of σ_1_Rs [159].

### 3.2. Relation of Gene Expression Effects to Behavioral Effects

The linkage of the molecular effects described above to behavioral outcomes is beginning to be elucidated. Romieu *et al.* [99] were the first to report changes in gene expression related to repeated cocaine treatment in a place conditioning procedure. In that study, repeated cocaine treatment increased expression of σ_1_Rs in the NAC, but not in the caudate putamen, prefrontal cortex, or cerebellum. This regional selectivity suggests the importance of the adaptive change in σ_1_Rs for the effects of cocaine in place conditioning.

Liu and Matsumoto [160] confirmed the increases in locomotor activity over 15 days of treatment with cocaine (10 mg/kg). The authors also examined fra-2 and σ_1_R gene expression (using RT-PCR) and proteins (Western blots). Along with locomotor activity, fra-2 and σ_1_R genes and proteins increased with cocaine exposure. Further, the σR antagonist, BD 1063, attenuated the gene and gene product changes as well as the locomotor sensitization produced by cocaine [160]. The authors concluded that repeated cocaine exposure produces increases in fra-2 and σ_1_Rs which underlie the sensitization produced.

The self administration of methamphetamine has also been shown to alter σ_1_R systems. In one study [161], rats self administered methamphetamine (0.1 mg/kg/injection) whereas one of two other groups received the same dose of methamphetamine with frequencies matched to the group that self administered the drug (yoked control). A second control group received saline injections with a matching frequency. Western blots revealed a 50% increase in σ_1_R protein in midbrain and Northern blots showed decreases in σ_1_R mRNA levels in frontal cortex and increases in hippocampus of subjects actively self administering methamphetamine, but not in the yoked methamphetamine-exposed or saline-control rats. The authors concluded that increases in σ_1_Rs contribute to the reinforcing effects of methamphetamine. A previous study [162] found a down-regulation of DA D2 autoreceptors with methamphetamine self administration. The authors speculated that the down-regulation of DA D2 autoreceptors would increase adenylate cyclase, and consequently protein kinase A (PKA) activity. As one part of this report the authors showed that dibutyryl cAMP which activates PKA increased σ_1_R protein level in NG-108 cells compared to vehicle exposed cells. Therefore, the results suggest that σ_1_R up-regulation, induced by methamphetamine self administration is mediated by increased PKA activity due to DA D2 autoreceptor down-regulation.

In a subsequent study [163], methamphetamine, whether self administered or passively received, significantly elevated σ_1_Rs, as well as the endoplasmic reticulum chaperones BiP and calreticulin in the ventral tegmental area and substantia nigra. In the olfactory bulb, however, only the σ_1_R chaperone was increased, and this increase occurred only in rats that actively self administered methamphetamine. Also in the olfactory bulb σ_1_Rs were co-localized with DA D1 receptors. The authors concluded that methamphetamine whether administered actively or passively induced ER stress which precipitates the activity of ER chaperones. However, the changes seen only in rats that actively self administered methamphetamine suggests that D1 and σ_1_Rs in the olfactory bulb might play an important role in the self administration of methamphetamine. The relation between DA D1 and σ_1_Rs in the olfactory bulb is particularly interesting in light of findings that a D1 agonist, (+)-SKF 38393, is more effective than cocaine in regulating σ_1_Rs in SVG cells [164]. Curiously in that study σ_1_Rs were down- rather than up-regulated. Another linkage between D1 and σ_1_Rs comes from studies suggesting that these proteins form heterodimers [28]. Finally, the up-regulation of σ_1_Rs by cocaine administration *in vivo* does not occur in mice with a genetic deletion of DA D1 receptors [165].

## 4. Summary and Conclusions

As the studies of gene regulation demonstrate, there is substantial evidence of the involvement of σ_1_Rs in behavioral effects of stimulant drugs. Studies of the time course of effects suggest specific changes in fra-2 are followed by an up regulation of σ_1_Rs, and that those changes are reasonably well correlated with behavioral changes. Causality on the other hand is obviously difficult to conclusively demonstrate. The changes in behavior occur exceedingly rapidly. As a consequence, improved time resolution of demonstrated genetic changes may help determine which brain regions are the most critical for the demonstrated effects. In addition, the application of technological advances allowing direct manipulation of genetic changes will provide tests of hypotheses regarding the importance of the regulation of particular genes. As the first section of this paper shows, substantial and significant advances have accrued in the understanding of the cellular biology of σRs. These advances provide a blue print for further studies that are focused on the cellular mechanisms involved in the changes that accompany exposure to stimulant drugs.

It is likely important to note that the most studied (with regard to σR influence) behavioral outcomes of stimulant administration are locomotor activity and place conditioning. These procedures have exhibited a remarkable similarity in their σR pharmacology. In particular, the behavioral effects of stimulants under both procedures are sensitive to σR antagonism. This sensitivity appears to be extant in both the acquisition and the manifestation of stimulant-induced locomotor sensitization and place conditioning. Further, while σR antagonists can block the effects of stimulants, σR agonists are inactive on their own, though may increase the effects of stimulants. On the other hand, the σR pharmacology of stimulant self administration has been less well studied. However, current evidence suggests that it is different from what has been elucidated so far with the other behavioral procedures. For example, cocaine self administration is not altered by σR antagonist administration, whereas both place conditioning and sensitized locomotor activation are readily blocked by σR antagonists. Whether the acquisition of cocaine self administration is sensitive to σR antagonism has not yet been determined. In contrast to the negative findings in place conditioning, σR agonists alone are self administered, and their administration also shifts the stimulant self-administration dose-effect curves leftward. The above differences in the outcomes with different behavioral procedures indicate that the resultant effects do not represent interchangeable readouts of a singular neurobiological mechanism.

Clearly a more thorough characterization of the behavioral and cellular pharmacology of σR agonists and antagonists, both alone and in combination with stimulant drugs, will help to elucidate the role of σRs in the behavioral changes that accompany exposure to stimulant drugs. As we delve more deeply into the σR cellular mechanisms involved in the effects of stimulant drugs, it is important to remain cognizant of the differences in behavioral outcomes which point to important differences in their underlying mechanisms which may have implications for an ultimate application of this knowledge in the development of medications to treat stimulant abuse.

## Figures and Tables

σ_1_Rs are ER proteins highly clustered at the mitochondria-associated ER membrane (MAM). Under un-stressed conditions, σ_1_Rs regulate IP3 receptor-mediated Ca2+ mobilization at the MAM. Ca2+ directly influxed from MAM to mitochondria activates ATP production. When σ_1_Rs at the MAM are depleted in neurons, it causes various mitochondrial dysfunctions, including aggregated mitochondria, membrane potential changes and increased ROS production. These are followed by cytochrome c release and the activation of caspase-3. The activated caspase-3 induces the cleavage and inactivation of the Rac 1 specific GEF Tiam1, leading to the failure of Rac GDP switching to Rac GTP. The failure of the Tiam1-Rac pathway causes the disrupted cytoskeleton network and actin polymerization, that partly involves reduced activities of capping proteins and branching proteins Arp2/3. Consequently, neurons depleted with σ_1_Rs fail to promote maturation of dendritic spines and recruitment of AMPA/NMDA to post-synaptic membranes as evident of those receptors remained in the dendritic shaft. σ_1_Rs may also modulate post-synaptic proteins including a variety of ion channels (Na2+, K+ channels) and G protein-coupled receptors (DA D2, μ opioid receptors) by translocating to the proximity of postsynaptic density.

The tissue was incubated in 50 mM Tris-HCl buffer, pH 8.0 with 3.0 nM [^3^H]DTG for 120 min at room temperature. Each tube contained 3 nM [^3^H]DTG (specific activity 48 Ci/mmol), 200 nM (+)-pentazocine, and 8.0 mg tissue. Non-specific binding was defined by addition of 10 μM haloperidol. Reaction was terminated by filtration through Whatman GFB filters presoaked in 0.05% polyethylenimine. The curve is representative of three independent experiments carried out in triplicate.

Increased levels of DA were expressed as the average percent change in basal DA values during the first 30 min after DTG, PRE-084, or cocaine treatment. The top panel shows effects of pretreatments with saline, 1 mL/kg i.p.; BD 1063 (a preferential σ_1_R antagonist), 10 and 30 mg/kg i.p.; BD 1008 (non-selective σR antagonist), 10 mg/kg i.p.; and SN 79 (preferential σ_2_R antagonist), 1 and 3 mg/kg i.p., injected 30, 30, 30, 15 and 15 min, respectively, before DTG administration, 5.6 mg/kg i.v. The middle panel shows effects of pretreatments with saline, 1 mL/kg i.p.; BD 1063, 10 mg/kg i.p.; and BD 1008, 10, and 30 mg/kg i.p., injected 30 min before PRE 084 administration, 10.0 mg/kg i.v. The bottom panel shows effects of pretreatments with saline, 1 mL/kg i.p.; BD 1063, 10 mg/kg i.p.; and BD 1008, 10 mg/kg i.p. injected 30 min before cocaine administration, 0.32 mg/kg i.v. The data shown are published results [56], and the reader is referred to that paper for more details and information about the original experiments. concomitant with stimulant self administration [83] these findings have clear implications for stimulant dependence.

Ordinates: Responses per sec. Abscissae: Dose of cocaine or each substituted drug in mg/kg/injection. Each point represents the mean ± SEM (N = 6). EXT: extinction (responses have no consequences). Panel A: Effects of pre-session treatments with the σR antagonist, BD 1063 (3.2, 10 and 32 mg/kg, i.p., 5 min before sessions) on cocaine self administration; Panel B: Effects of pre-session treatments with the σR agonist PRE-084 (1.0, 3.2 and 10 mg/kg, i.p., 30 min before sessions) on cocaine self administration. Panel C: Self administration of the σR agonists PRE-084 (open circles) and DTG (open triangles up) when substituted for cocaine.

## Appendix 1: Non-Standard Abbreviations and Relevant Compounds

4-IBP: 4-(N-benzylpiperidin-4-yl)-4-iodobenzamide

(+)-MR200: (+)-methyl 2-[[4-(4-chlorophenyl)-4-hydroxypiperidin-1-yl]methyl]-1-phenylcyclopropanecarboxylate oxalate

(+)-3-PPP: 3-(3-hydroxyphenyl)-N-*n*-propylpiperone HCl

AC 927: N-phenethylpiperidine oxalate

BD 1008: N-[2-(3,4-dichlorophenyl)ethyl]-N-methyl-2-(1-pyrrolidinyl)ethylamine dihydrobromide

BD 1047: N-[2-(3,4-dichlorophenyl)ethyl]-N-methyl-2-(dimethylamino)ethylamine dihydrobromide

BD 1063: 1-[2-(3,4-dichlorophenyl)ethyl]-4-methylpiperazine dihydrochloride

BMY 14802: α-(4-fluorophenyl)-4-(5-fluoro-2-pyrimidinyl)-1-piperazinebutanol

BTCP: N-[1-(2-benzo(b)thiophenyl) cyclohexyl]piperidine

Ca2+: Calcium ion

DA: dopamine

DAT: dopamine transporter

DHEA: dehydroepiandrosterone (3β-hydroxy-5-androsten-17-one), σR agonist

DTG: 1,3-di-*o*-tolylguanidine

ER: endoplasmic reticulum

EXT: extinction

FR: fixed ration

igmesine: σR agonist

Inj: injection

LR172: N-[2-(3,4-dichlorophenyl)ethyl]-N-methyl-2-(1-homopiperidinyl)ethylamine

MAM: mitochondria-associated ER membrane

MS-377: (R)-(+)-1-(4-chlorophenyl)-3-[4-(2-methoxyethyl)piperazin-1-yl]methyl-2- pyrrolidinone L-tartrate

NAC: nucleus accumbens

NE: norepinephrine

NE-100: *N*,*N*-dipropyl-2-[4-methoxy-3-(2-phenylethoxy)phenyl]ethylamine monohydrochloride

NET: norepinephrine transporter

PKA: protein kinase A

PCP: phencyclidine

PRE-084: 2-(4-Morpholinethyl) 1-phenylcyclohexane-1-carboxylate hydrochloride

Pregnenolone: 3β-hydroxy-5-pregnen-20-one, σR agonist

Progesterone: 4-pregnene-3,20-dione, σR antagonist

Rimcazole: 9-[3-(cis-3,5-dimethyle-1-piperazinyl)propyl]-9H-carbazole dihydrochloride

SERT: serotonin transporter

SKF 10,047: *N*-allylnormetazocine

WIN 35,428: (-)-2ß-carbomethoxy-3ß-(4-fluorophenyl)tropane

σ_1_R: Sigma 1 receptor

σ_2_R: Sigma 2 receptor

σR: Sigma receptor
